# Tetra-ARMS PCR analysis of angiotensinogen AGT T174M (rs4762) genetic polymorphism in diabetic patients: a comprehensive study

**DOI:** 10.3389/fendo.2023.1240291

**Published:** 2023-08-25

**Authors:** Muhammad Sajid Hamid Akash, Momina Shahid, Shaleem Suhail, Kanwal Rehman, Ahmed Nadeem, Tahir Maqbool Mir

**Affiliations:** ^1^ Department of Pharmaceutical Chemistry, Government College University, Faisalabad, Pakistan; ^2^ Department of Pharmacy, The University of Faisalabad, Faisalabad, Pakistan; ^3^ Department of Pharmacy, University of Chenab, Gujrat, Pakistan; ^4^ Department of Pharmacy, The Women University, Multan, Pakistan; ^5^ Department of Pharmacology and Toxicology, College of Pharmacy, King Saud University, Riyadh, Saudi Arabia; ^6^ National Center for Natural Products Research, School of Pharmacy, University of Mississippi, Oxford, MS, United States

**Keywords:** AGT gene polymorphism, hypertension, hypertensive diabetic, Tetra-ARMS PCR, genotyping

## Abstract

**Background and purpose:**

Hypertension (HTN) is a multifactorial chronic disease that poses a significant global health burden and is associated with increased mortality rates. It often coexists with other conditions, such as cardiovascular, liver, and renal diseases, and has a strong association with diabetes mellitus. Insulin resistance and endothelial dysfunction commonly occur in individuals with both HTN and type 2 diabetes mellitus (T2DM). Genetic factors, along with environmental and pathological factors, play a role in the development of HTN. Recent studies have revealed the influence of single nucleotide polymorphisms (SNPs) in various genes on HTN. In this study, we aimed to investigate the genetic polymorphism of angiotensinogen (AGT) T174M (rs4762) and its association with HTN in diabetic patients.

**Methods:**

A total of 300 participants were enrolled in this study and divided into three groups: control, hypertensive, and hypertensive diabetic. Blood samples were collected, and predetermined biochemical parameters were assessed. Genotyping of the AGT T174M (rs4762) gene was conducted using Tetra ARMS PCR with specific primers.

**Results:**

The study findings revealed a significant association between AGT T174M (rs4762) genotype and HTN in diabetic patients within the Pakistani population. The C/T genotype of AGT T174M (rs4762) was found to be significant in both the hypertensive and hypertensive diabetic participants compared to the control group. This genotype was identified as a risk factor for developing HTN in both the hypertensive and hypertensive diabetic participants.

**Conclusion:**

This study demonstrates a significant association between AGT T174M (rs4762) genetic polymorphism and HTN in diabetic patients. The C/T genotype of AGT T174M (rs4762) may serve as a potential marker for identifying individuals at risk of developing HTN, specifically in the hypertensive and hypertensive diabetic populations. Further research is warranted to elucidate the underlying mechanisms and validate these findings in larger cohorts.

## Introduction

Hypertension (HTN) stands as one of the primary causes of deaths related to cardiovascular diseases (CVDs), greatly increasing the risk of conditions such as stroke, heart attack, coronary artery disease (CAD), renal diseases, diabetes mellitus (DM), and other associated disorders (1–5). Individuals with pre-existing DM have a 50% higher risk of developing HTN, while the chances of developing CVDs increase 4-5 times in patients with both HTN and DM (2, 6). Research has demonstrated that traditional risk factors, including diet, obesity, insulin resistance, high blood pressure, hyperlipidemia, inflammatory responses, tobacco use, and excessive alcohol consumption, can roughly predict 50% of a patient’s risk of experiencing a CVD episode (7). The strong association between HTN, DM, and CVDs is also supported by various genome-wide association studies (8). Several factors contribute to the development and pathogenesis of HTN, with environmental factors, fatty diets, high salt intake, lack of physical activity, and stress being among the most crucial ones (9, 10). Additionally, the prevalence of HTN varies across different ethnicities (9, 11). Thus, racial and gender differences play a significant role in the relationship between the development of insulin resistance and DM associated HTN (2, 9, 11, 12). Studies have also reported that diabetic females with impaired glucose tolerance are more susceptible to developing HTN compared to males with similar impairments in glucose homeostasis (13).

Insulin resistance plays a pivotal role in the association between DM and HTN. HTN is prevalent in diabetic patients, with approximately 50-80% of individuals with type 2 diabetes mellitus (T2DM) affected, indicating the crucial involvement of insulin resistance in HTN development (14–16). Elevated levels of insulin contribute to increased sodium reabsorption, renin excretion, and sympathetic activity, ultimately leading to high blood pressure (17). Literature further supports the notion that HTN exacerbates the risk and predisposition to DM and its associated diseases, resulting in heightened comorbidity and mortality compared to non-hypertensive patients (1, 2, 4, 16, 18, 19).

In addition to various physical and environmental factors, significant attention is now being directed towards assessing the genetic causes of this disease. Single Nucleotide Polymorphism (SNP) involves the substitution of a single nucleotide within the genetic material and account for over 90% of genetic polymorphisms (20). Such alterations can lead to changes in enzyme activity due to amino acid sequence modifications, affecting transcription, intrinsic termination, and factor-dependent termination. However, in some cases, SNPs or mutations may not impact enzyme activities, underscoring the importance of identifying those mutations that specifically affect gene function (21).

The renin-angiotensin-aldosterone system (RAAS) has recently garnered significant attention due to its potential to induce insulin resistance, ultimately leading to DM. It plays a major role in the pathogenesis of HTN, insulin resistance, and subsequently, DM. Among the components of RAAS, angiotensin (AGT) holds particular significance (22–25). AGT plays a crucial role in the synthesis of AGT I and II, with an essential impact on blood pressure regulation through the regulation of sodium excretion (26). Renin, released from the kidney, acts on AGT released by the liver, leading to the formation of AGT I. Angiotensin Converting Enzyme (ACE), released from the lungs, converts Angiotensin I to Angiotensin II (an octapeptide). Angiotensin II plays a vital role in regulating hypertension through vasoconstriction and sodium-water retention (27). Various polymorphic forms of AGT have been identified, demonstrating evidence of association with hypertension and certain CVDs. A recent study highlighted the involvement of AGT (rs699) in insulin sensitivity, while AGT rs4762 and rs699 have shown significant associations with hypertension in several ethnic populations (28, 29). In this study, we aimed to analyze the association between genetic polymorphism of AGT T174M (rs4762) and HTN in the Pakistani population. Additionally, we investigated the prevalence of genetic polymorphism of AGT T174M (rs4762) in HTN with the presence of DM as a risk factor, comparing it with healthy individuals.

## Materials and methods

### Ethical considerations

This study received approval from the Ethical Review Committee (Ref. No. GCUF/ERC/39) of Government College University Faisalabad (GCUF). Prior to the initiation of blood sample collection, informed written consent was obtained from all the participants or their respective guardians.

### Study design

This present study aimed to assess the biochemical association between the genetic polymorphism of AGT T174M (rs4762) in hypertensive and hypertensive-diabetic patients. Blood sampling was conducted at Allied Hospital, Faisalabad, and Punjab Employees Social Security Institute, Faisalabad, Pakistan in accordance with the approved guidelines provided by the ethical committee of GCUF, Pakistan. A total of 300 study subjects were selected for blood sampling and divided into three groups. Group I consisted of control participants (n=100), Group II included HTN patients (n=100), and Group III comprised hypertensive-diabetic (HTN-DM) patients (n=100). Informed written consent was obtained from each patient, and a comprehensive questionnaire was completed to gather necessary information about the study participants. Blood samples were collected from the target population and subjected to biochemical analysis. Genotyping, using Tetra-ARMS PCR, was performed to analyze the genetic polymorphism among HTN and HTN-DM patients.

### Inclusion and exclusion criteria

Participants between the ages of 30 and 70 years were selected for this study. Individuals below the age of 30 or above the age of 70 were excluded. Participants with a history of prolonged medication use for comorbidities, any other medical conditions, alcohol consumption, or any relevant medical history were also excluded. Standard blood pressure measurements were employed for diagnosing HTN and HTN-DM patients. HTN-DM patients included in the study had an HbA1c value higher than 5.7%. Similarly, HTN patients were receiving antihypertensive treatment, including ACE inhibitors such as lisinopril and captopril, angiotensin receptor blockers (ARBs) like candesartan and losartan, β-blockers such as metoprolol and atenolol, calcium channel blockers like verapamil and amlodipine, as well as diuretics such as furosemide and hydrochlorothiazide and statins such as atorvastatin. HTN-DM patients were also receiving antidiabetic medications, including insulin, glipizide, and metformin, in addition to antihypertensive treatment.

### Measurement of blood pressure

The blood pressure of the participants was measured randomly using a digital BP apparatus. Hypertensive patients exhibited systolic blood pressure equal to or greater than 140 mmHg and diastolic blood pressure equal to or greater than 90 mmHg.

### Collection of blood samples

Approximately 5-6 ml of blood was collected from each participant and evenly distributed into two separate tubes. 2.5-3 ml of blood was placed in EDTA tubes, while another 2.5-3 ml was collected in a vacutainer containing a gel clot activator. An icebox or ice packs were prepared to maintain the blood sample’s temperature during transportation from the hospital to the laboratory. The collected blood sample was subjected to centrifugation to separate the blood serum, which was subsequently stored at -20°C for further biochemical analysis.

### Analysis of biochemical parameters

The serum, obtained from the blood samples, underwent examination to assess multiple biochemical parameters, including the glycemic profile [Random Blood Sugar (RBS), Hemoglobin A1c (HbA1c)], liver biomarkers [Alanine transaminase (ALT), Alkaline phosphatase (ALP)], renal biomarkers [urea, creatinine, and uric acid], lipid profile [cholesterol, triglycerides], and albumin levels. This analysis was conducted using specific kits and a biochemical analyzer (Microlab-300) for accurate measurement.

### Genotype Analysis

#### Extraction of DNA

The collected blood sample underwent manual processing to extract genomic DNA. Blood was collected in Eppendorf tubes and mixed with RBC lysis buffer. The mixture was then shaken and centrifuged at 7000 rpm for 2 minutes. The resulting pellets were disrupted using a vortex mixer and rinsed with RBC lysis buffer again. Saturated NaCl (5M), chloroform, and nucleic acid lysis buffer were added to the Eppendorf tube, mixed, and centrifuged at 7000 rpm for 2 minutes. After transferring the supernatant to a fresh Eppendorf tube, cold ethanol was added, and the mixture was centrifuged at 12000 rpm for 1 minute. The supernatant was discarded, and the remaining DNA pellet was vortexed after the addition of TE buffer. The Eppendorf tube containing the DNA was stored at -20°C for genotyping purposes.

#### Analysis of DNA

The NanoDrop method and gel electrophoresis were employed for the quantitative and qualitative analysis of DNA, respectively. The purity of the extracted DNA was assessed by loading the samples onto a 2% agarose gel.

#### Genotyping of AGT gene (rs 4762)

Tetra-ARMS PCR was used to genotype the SNP of the AGT T174M (rs4762) gene. The amplification of the AGT T174M (rs 4762) gene was performed using two forward primers and two reverse primers, as outlined in **Table 1**. The PCR process was performed using the “Thermocycler Master Cycler Gradient.” The initial denaturation step was carried out at 94°C for 10 minutes. Subsequently, denaturation was performed at 94°C for 1 minute, followed by annealing at 65°C for 1 minute, and extension at 72°C for 1 minute. This cycle was repeated for a total of 30 times starting from step 2. The final extension step was set at 72°C for approximately 5 minutes, as shown in **Table 2**. Following the completion of the PCR reaction, 20 μL of the resulting product was loaded onto a 2% agarose gel, stained with ethidium bromide, submerged in TAE buffer, and subjected to electrophoresis in an electric field. The gel was subsequently visualized under UV light using a gel documentation system.

**Table 1:** The Primer sequence of AGT T174M (rs4762).

Gene primers	Primer sequence
Forward inner primer	5’GCCCAGCTGCTGCTGTCAAC3
Forward outer primer	5’TTCCGTATATATGGCATGCACAGTGA3’
Reverse inner primer	5’TGTGAACACGCCCACCAACA3’
Reverse outer primer	5’GAGCAGCCAGTCTTCCATCCTGT3’

**Table 2:** Tetra-ARMS PCR temperatures, time and number of cycles of AGT T174M (rs4762).

Reaction Condition	Temperature	Time
Initial Denaturation	94	10 min
Denaturation	94	1 min
Annealing	65	1 min
Extension	72	1 min
No. of cycles	30	–
Final extension	72	5 min

### Statistical analysis

Statistical analysis was performed using SPSS, GraphPad Prism 5, and Minitab. Significant differences and the genotype frequency of AGT T174M (rs4762) among the different study groups were assessed using one-way ANOVA, Tukey’s multiple comparison test, and Fisher’s exact test.

## Results

### Analysis of demographic and clinical parameters

In the hypertensive study group, females accounted for 37% while males accounted for 26%. In the hypertensive-diabetic study group, females accounted for 28% while males accounted for 45%. The mean values of systolic blood pressure were 125.2 ± 7.7 mmHg, 169.5 ± 30.87 mmHg, and 167.8 ± 17.9 mmHg for the control, hypertensive, and hypertensive-diabetic study groups, respectively. The mean values of diastolic blood pressure were 82.7 ± 3.14 mmHg, 96.33 ± 9.45 mmHg, and 97.38 ± 7.45 mmHg for the control, hypertensive, and hypertensive-diabetic study groups, respectively (**Table 3**).

**Table 3:** Demographic and Clinical parameters among control, hypertensive and hypertensive-diabetic study groups.

Characteristics	Control	Hypertensive	Hypertensive-Diabetic
Gender wise distribution (Sex)			
Females	70 (37%)	68 (37%)	50 (26%)
Males	30 (27%)	32 (28%)	51 (45%)
Age (years)			
Less than 50 years	65%	57%	43%
Greater than 50 years	35%	43%	57%
Blood pressure (mmHg)			
Systolic BP	125.2 ± 7.7	169.5 ± 30.87	167.8 ± 17.9
Diastolic BP	82.7 ± 3.14	96.33 ± 9.45	97.38 ± 7.45
No. of Participants	100	100	100

### Biochemical analysis

The glycemic profile, represented by RBS and HbA1c, was significantly higher in the hypertensive and hypertensive-diabetic groups compared to the control group. The liver biomarkers, including ALT and ALP, were significantly elevated in the hypertensive and hypertensive-diabetic study groups compared to the controls. The renal biomarkers, including urea and uric acid, were also significantly higher in the hypertensive and hypertensive-diabetic study groups compared to the controls, while creatinine levels were significantly lower in the hypertensive and hypertensive-diabetic study groups compared to the control group. The lipid profile, including cholesterol levels, was significantly elevated in the hypertensive and hypertensive-diabetic study groups compared to the controls, whereas triglyceride levels did not show a significant difference between the hypertensive and hypertensive-diabetic groups and the controls. Furthermore, albumin levels were significantly lower in the hypertensive and hypertensive-diabetic study groups compared to the controls (**Table 4**).

**Table 4:** Biochemical analysis of control, hypertensive and hypertensive-diabetic study groups.

Biochemical test	Control	Hypertensive	Hypertensive- Diabetic	*P*- value
Random blood sugar	135.0 ± 10.68	157.0 ± 26.09	260.1 ± 40.89	< 0.05
Hemoglobin A1c	4.744 ± 0.403	5.227 ± 0.5421	7.057 ± 0.3718	< 0.05
Alanine Transaminase	28.51 ± 9.144	37.17 ± 9.520	46.37 ± 12.43	< 0.05
Alkaline Phosphatase	241.9 ± 48.30	271.9 ± 57.44	291.6 ± 37.61	< 0.05
Urea	34.02 ± 5.181	42.59 ± 22.27	52.71 ± 20.17	< 0.05
Uric acid	5.219 ± 1.583	6.123 ± 2.808	7.437 ± 1.544	< 0.05
Creatinine	1.374 ± 0.464	1.213 ± 1.289	1.186 ± 0.579	>0.05
Cholesterol	176.7 ± 16.20	201.0 ± 24.97	192.6 ± 22.19	< 0.05
Triglycerides	154.2 ± 10.73	156.5 ± 18.55	153.2 ± 21.32	>0.05
Serum Albumin	4.430 ± 0.6141	3.387 ± 0.5171	2.412 ± 0.3493	< 0.05

### Genotyping

AGT T174M (rs 4762) genotyping was performed on all subjects in the control, hypertensive, and hypertensive-diabetic study groups. The distribution of allelic and genotype frequencies was analyzed using SNP Stat software (http://bioinfo.iconcologia.net/SNPstats), a tool commonly employed for evaluating genetic models.

### Comparative analysis of allelic frequency

The allelic frequency distribution of the C and T alleles among the control, hypertensive, and hypertensive-diabetic groups clearly indicates that the C allele was more prevalent in the hypertensive and hypertensive-diabetic groups compared to the controls (**Table 5**).

**Table 5:** Allele and genotype frequencies of AGT gene rs4762 between control, hypertensive and hypertensive-diabetic study groups.

Groups	Allele		Genotype			*P*-value
	C	T	C/C	C/T	T/T	
Control	155	90	156	38	6	0.04
Hypertensive	170	60	74	110	16	
Hypertensive-diabetic	185	30	68	120	12	

### Comparative analysis of genotype frequency

Fisher’s exact test was utilized for the analysis of Hardy-Weinberg equilibrium to determine the genotype frequency. The genotype frequency of AGT T174M (rs4762) in the control, hypertensive, and hypertensive-diabetic study groups revealed a higher occurrence of the C/T genotype among the hypertensive and hypertensive-diabetic groups compared to the controls (**Table 5**).

### Comparative analysis of single nucleotide polymorphism

The analysis of SNP among the control, hypertensive, and hypertensive-diabetic groups revealed significant differences for the heterozygous genotype C/T under the codominant [OR=4.30, 95% CI=2.24-8.25, P<0.05], dominant [OR=4.70, 95% CI=2.54-8.71, P<0.05], and overdominant [OR=3.49, 95% CI=1.85-6.59, P<0.05] genetic models between the control and hypertensive groups (**Table 6**). Additionally, a significant difference was observed for the heterozygous genotype C/T under the codominant [OR=6.48, 95% CI=3.40-12.36, P<0.05], dominant [OR=5.78, 95% CI=3.11-10.78, P<0.05], and overdominant [OR=6.39, 95% CI=3.37-12.13, P<0.05] genetic models between the control and hypertensive-diabetic groups (**Table 7**). Moreover, a significant difference was found for the heterozygous genotype C/T under the codominant [OR=0.19, 95% CI=0.04-0.90, P<0.05], recessive [OR=0.15, 95% CI=0.03-0.69, P<0.05], and overdominant [OR=1.83, 95% CI=1.05-3.21, P<0.05] genetic models between the hypertensive and hypertensive-diabetic study groups (**Table 8**).

**Table 6:** Association analysis of AGT gene rs4762 in control and hypertensive study groups.

Genetic model	Genotype	Control	Hypertensive	OR (95% CI)	*P*-value
Codominant	C/C	156 (78%)	86 (43%)	1.00	<0.0001
	C/T	38 (19%)	90 (45%)	4.30 (2.24-8.25)	
	T/T	6 (3%)	24 (12%)	7.26 (1.94-27.13)	
Dominant	C/C	156 (78%)	86 (43%)	1.00	<0.0001
	C/T-T/T	44 (22%)	114 (57%)	4.70 (2.54-8.71)	
Recessive	C/C-C/T	194 (97%)	176 (88%)	1.00	0.013
	T/T	6 (3%)	24 (12%)	4.41 (1.20-16.14)	
Overdominant	C/C-T/T	162 (80.7%)	110 (55%)	1.00	<0.0001
	C/T	38 (19.3%)	90 (45%)	3.49 (1.85-6.59)	

**Table 7:** Association analysis of AGT gene rs4762 in control and hypertensive-diabetic study groups.

Genetic model	Genotype	Control	Hypertensive-Diabetic	OR (95% CI)	*P*-value
Codominant	C/C	158 (79%)	76 (38%)	1.00	0.0001
	C/T	38 (19%)	120 (60%)	6.48 (3.40-2.36)	
	T/T	6 (3%)	4 (2%)	1.37 (0.22-8.54)	
Dominant	C/C	156 (78%)	76 (38%)	1.00	0.0001
	C/T-T/T	44 (22%)	124 (62%)	5.78 (3.11-0.78)	
Recessive	C/C-C/T	194 (97%)	196 (98%)	1.00	0.65
	T/T	6 (3%)	2 (2%)	0.66 (0.11-4.04)	
Overdominant	C/C-T/T	162 (81%)	80 (40%)	1.00	0.0001
	C/T	38 (19%)	120 (60%)	6.39 (3.37-2.13)	

**Table 8:** Association analysis of AGT gene rs4762 in hypertensive and hypertensive-diabetic study groups.

Genetic model	Genotype	Hypertensive	Hypertensive-Diabetic	OR (95% CI)	*P*-value
Codominant	C/C	86 (43%)	76 (38%)	1.00	0.0056
	C/T	90 (45%)	120 (60%)	0.19 (0.04-0.90)	
	T/T	24 (12%)	4 (2%)	1.51 (0.840-2.70)	
Dominant	C/C	86 (43%)	76 (38%)	1.00	0.47
	C/T-T/T	114 (57%)	124 (62%)	1.23 (0.70-2.17)	
Recessive	C/C	176 (88%)	196 (98%)	1.00	0.0036
	C/T-T/T	24 (12%)	4 (2%)	0.15 (0.03-0.69)	
Overdominant	C/C-T/T	110 (55%)	80 (40%)	1.00	0.033
	C/T	90 (45%)	120 (60%)	1.83 (1.05-3.21)	

### Clinical and biochemical association of AGT T174M (rs 4762)

Systolic blood pressure was higher in the hypertensive and diabetic hypertensive groups compared to the control group. Furthermore, the mean of the heterozygous genotype C/T was higher in the hypertensive and diabetic hypertensive groups than in the homozygous genotype’s C/C and T/T. Additionally, diastolic blood pressure was higher in the hypertensive and diabetic hypertensive study groups compared to the control group, and the mean of the heterozygous genotype C/T was higher than that of the homozygous genotype’s C/C and T/T. The overall p-value was also significant, indicating an association between the C/T genotype and the development of hypertension.

The levels of RBS and HbA1c were higher in the hypertensive and diabetic hypertensive groups compared to the control group, and the mean of the heterozygous genotype C/T was higher in the hypertensive and diabetic hypertensive groups compared to the control group. The levels of liver biomarkers, ALT, and ALP were higher in the hypertensive and diabetic hypertensive groups compared to the control group, and the mean of the heterozygous genotype C/T was higher in the hypertensive and diabetic hypertensive groups. The renal biomarkers, including urea, and uric acid, were higher in the hypertensive and diabetic hypertensive groups compared to the control group, and the mean of the heterozygous genotype C/T was higher in the hypertensive and diabetic hypertensive group. However, creatinine was lower in the hypertensive and diabetic hypertensive groups compared to the control group, and the mean of the heterozygous genotype C/T was lower in the hypertensive and diabetic hypertensive study groups.

The lipid profile, specifically cholesterol, was higher in the hypertensive and diabetic hypertensive groups compared to the control group, and the mean of the heterozygous genotype C/T was higher in the hypertensive and diabetic hypertensive study groups. On the other hand, triglycerides were lower in the hypertensive and diabetic hypertensive study groups compared to the controls, and the mean of the heterozygous genotype C/T was lower in the hypertensive and diabetic hypertensive study groups (**Table 9**).

**Table 9:** The Clinical and Biochemical data for control, hypertensive, and hypertensive-diabetic study groups according to Genotype (C/C, C/T, T/T).

Parameters	Controls			Hypertensive			Hypertensive-Diabetic			*P*-value
	C/C	C/T	T/T	C/C	C/T	T/T	C/C	C/T	T/T	
Clinical										
Systolic BP	124.7 ± 7.862	127.7 ± 7.198	120.0 ± 0.0	172.7 ± 36.19	168.0 ± 15.81	160.0 ± 15.81	163.0 ± 15.85	172.2 ± 19.16	170.0 ± 0.0	< 0.05
Diastolic BP	82.67 ± 3.126	82.73 ± 3.438	85.00 ± 0.0	97.03 ± 10.99	96.05 ± 8.555	94.00 ± 5.477	95.73 ± 7.120	98.84 ± 7.625	100.0 ± 0.0	< 0.05
Biochemical										
Random Blood Sugar	134.4 ± 10.96	136.4 ± 9.92	144.0 ± 0.0	159.9 ± 35.50	156.6 ± 15.10	141.0 ± 18.12	252.2 ± 48.98	266.3 ± 30.96	289.0 ± 1.414	< 0.05
Hemoglobin A1c	4.758 ± 0.407	4.736 ± 0.388	4.200 ± 0.0	4.903 ± 0.418	4.903 ± 0.418	5.680 ± 0.497	6.973 ± 0.389	7.119 ± 0.337	7.450 ± 0.353	< 0.05
Alanine Transaminase	27.49 ± 9.324	31.73 ± 7.656	39.00 ± 0.0	38.16 ± 12.69	35.92 ± 6.470	40.40 ± 2.302	45.15 ± 14.37	47.40 ± 10.47	49.50 ± 12.05	< 0.05
Alkaline Phosphatase	237.3 ± 53.22	260.5 ± 11.05	244.0 ± 0.0	284.0 ± 79.73	261.1 ± 31.09	277.0 ± 22.29	279.2 ± 38.80	303.6 ± 33.43	287.0 ± 24.04	< 0.05
Protein urea	33.80 ± 5.025	34.00 ± 5.385	44.00 ± 0.0	50.59 ± 31.67	36.11 ± 7.210	40.60 ± 1.140	50.90 ± 20.68	54.51 ± 20.17	51.00 ± 11.31	< 0.05
Uric acid	5.349 ± 1.631	4.845 ± 1.344	3.500 ± 0.0	7.384 ± 3.669	5.108 ± 1.405	5.760 ± 0.826	7.656 ± 1.637	7.265 ± 1.437	6.650 ± 2.051	< 0.05
Serum Creatinine	1.247 ± 0.44	1.418 ± 0.560	0.900 ± 0.0	1.434 ± 1.930	0.873 ± 0.192	0.880 ± 0.258	1.293 ± 0.515	1.488 ± 0.616	1.100 ± 0.989	>0.05
Cholesterol	176.7 ± 17.02	175.2 ± 13.24	190.0 ± 0.0	204.6 ± 28.31	199.7 ± 22.95	188.4 ± 11.59	186.7 ± 15.45	197.7 ± 26.43	205.0 ± 9.899	<0.05
Triglycerides	154.6 ± 10.83	153.2 ± 10.77	154.0 ± 0.0	143.0 ± 20.20	149.9 ± 17.24	142.0 ± 14.75	145.0 ± 17.87	150.6 ± 24.24	145.5 ± 14.85	>0.05
Serum Albumin	4.416 ± 0.593	4.567 ± 0.687	3.600 ± 0.0	3.259 ± 0.597	3.584 ± 0.420	3.060 ± 0.270	2.429 ± 0.341	2.74 ± 0.351	2.50 ± 0.212	< 0.05

The data are presented as means ± SD.

## Discussion

AGT is an enzyme within the RAAS, playing a crucial role in blood pressure regulation (30). The RAAS system not only has a significant impact on blood pressure regulation but also influences volume homeostasis and renal hemodynamics (31). Disruptions in renal function resulting from AGT concentration changes can lead to hypertension (32). Previous studies have explored the association between gene polymorphisms and various diseases, revealing the involvement of gene polymorphisms in disease progression (33, 34). In this study, we observed a significant decrease in creatinine levels in the hypertensive and hypertensive-diabetic study group compared to the control group. Similar findings have been reported in previous studies, where low creatinine levels were observed in hypertensive and diabetic patients (35, 36). However, higher serum creatinine levels in hypertensive patients may serve as a predictor for future cardiovascular diseases (37). Serum ALP levels were significantly elevated in the hypertensive and hypertensive-diabetic study groups compared to the control group. These results align with another study’s findings (38, 39). Increased serum ALP levels indicate liver damage associated with hypertension and can also serve as an indicator for diabetes and coronary heart disease (40, 41). Serum ALT levels were significantly higher in the hypertensive and hypertensive-diabetic group compared to the control group. These findings are consistent with previous studies (42, 43). This not only confirms that ALT is a potent marker for hypertension but also suggests an increased risk of cardiovascular diseases (44).

Serum uric acid levels are found to be elevated in the hypertensive and hypertensive diabetic groups compared to the control group. Similar findings have been reported in a previously conducted study, where serum uric acid levels were higher in hypertensive and diabetic patients (45, 46). The relationship between serum uric acid and hypertension is still controversial, but a significant body of evidence suggests its crucial role in exacerbating hypertension. Additionally, certain studies suggest its involvement in activating the Renin Angiotensin System (46, 47). This increase in serum uric acid could be attributed to abnormal lipid and glucose metabolism, which is associated with higher levels of serum uric acid (48). Blood urea levels are higher in the hypertensive and hypertensive-diabetic groups compared to the control group. Increased blood glucose levels can lead to nephropathy, resulting in decreased renal function and overall reduced efficiency of the kidneys in excreting excess urea from the body (49).

The serum albumin level is found to be lower in the hypertensive and hypertensive-diabetic group compared to the control group. Similar results have been obtained in previous studies. However, the association between HTN and serum albumin level has been poorly studied (50). Nonetheless, in individuals with diabetes, the serum albumin level is inversely proportional to the diabetic condition, as it tends to decrease in diabetes mellitus (51, 52). The protective effect of serum albumin is diminished in hypertensive and diabetic patients, further contributing to the development of diseases such as ischemic stroke, heart failure, and coronary artery disease (53). The serum cholesterol level is found to be higher in the hypertensive and hypertensive-diabetic study group compared to the control group. High cholesterol levels are associated with increased blood pressure (54). Moreover, elevated serum cholesterol can be a significant risk factor for coronary artery disease and stroke among hypertensive individuals (55, 56).

The genetic polymorphism of AGT T174M (rs4762) gene was investigated in this research. AGT rs4762 polymorphism results in the substitution of threonine with methionine at the 174 position in the amino acid sequence (57). This polymorphism in AGT can increase the activity of the RAAS, leading to vasoconstriction. AGT II triggers the release of more aldosterone, ultimately causing an increase in plasma volume and hypertension (58, 59). Recent studies have shown an association between AGT T174M (rs4762) polymorphism and hypertension (58). Several studies have supported this association. A case-control study in China involving 538 individuals demonstrated a positive association between hypertension and AGT T174M (rs4762) genetic polymorphism (60). In this study, we observed a similar association and evaluated the involvement of AGT T174M (rs4762) polymorphism in HTN in the presence of diabetes mellitus, a known risk factor. We assessed liver and renal biomarkers in our study, revealing significant differences among the three study groups. Our analysis demonstrated that the C/T genotype was significantly more prevalent in the hypertensive and hypertensive-diabetic groups compared to the control group. Specifically, we observed that among the study participants, the number of individuals with the C/T genotype was 19% in the control group, 55% in the hypertensive group, and 60% in the hypertensive-diabetic group. These findings indicate a significant difference in the prevalence of the C/T genotype among the control, hypertensive, and hypertensive-diabetic groups.

This study was conducted over a limited duration, which may have impacted the ability to capture long-term effects or changes over time. The sample size used in the study was relatively small, which could limit the generalizability of the findings to a larger population. This study was conducted within a specific population, which may restrict the applicability of the results to other demographic groups or regions. Due to resource constraints, certain aspects of the study, such as comprehensive laboratory testing or extensive data collection, were not feasible. These limitations should be taken into consideration when interpreting the findings and generalizing them to broader populations. Future studies with larger sample sizes, longer durations, and diverse populations are recommended to overcome these limitations and provide a more comprehensive understanding of the topic.

### Recommendations and future prospects

Allele-specific genotyping investigations to detect DNA alterations at the single nucleotide level are relatively rare in underdeveloped countries. In this study, we identified a single allelic-level genetic mutation in the AGT gene among the hypertensive population. Such findings highlight the importance of conducting more genetic studies in diverse populations to uncover potential genetic variations associated with HTN. Furthermore, pharmacogenomics studies, like ours, play a crucial role in identifying new drug targets and mitigating adverse drug effects. This approach has the potential to significantly impact patient outcomes by reducing morbidity and mortality rates, improving treatment efficacy, and lowering healthcare costs. Our research contributes to the growing field of precision medicine, which has garnered global interest due to its ability to minimize side effects, enhance treatment effectiveness, and decrease the likelihood of disease recurrence. The findings from this study emphasize the significance of incorporating genetic information into clinical practice, enabling personalized treatment strategies for patients based on their unique genetic profiles. Continued advancements in precision medicine will allow for more targeted and tailored interventions, ultimately leading to improved healthcare outcomes and better management of chronic diseases.

## Conclusion

In conclusion, this study examined the association between AGT T174M (rs4762) genetic polymorphism and hypertension, particularly in the presence of DM. The results demonstrated a higher prevalence of the C/T genotype in both the hypertensive and hypertensive-diabetic study groups compared to the control group. These findings suggest a potential role of AGT T174M (rs4762) polymorphism in the development of hypertension, especially in individuals with coexisting diabetes. The biochemical analysis revealed significant differences in various clinical parameters between the control, hypertensive, and hypertensive-diabetic groups. Elevated blood pressure, altered glycemic profile, liver and renal biomarkers, and dyslipidemia were observed in the hypertensive and hypertensive-diabetic study groups compared to the controls. These findings support previous research indicating the association of these parameters with hypertension and highlight their potential role in the pathogenesis of the disease. The study also emphasized the importance of conducting genetic studies in diverse populations to identify potential genetic variations associated with hypertension. Allele-specific genotyping investigations provide valuable insights into DNA alterations at the single nucleotide level, which can contribute to the development of personalized treatment strategies and the advancement of precision medicine. However, it is important to acknowledge the limitations of this study, including the small sample size, limited duration, and specific population studied. Future research with larger sample sizes, longer durations, and diverse populations is recommended to validate and expand upon these findings. Overall, the findings from this study contribute to our understanding of the genetic and biochemical factors associated with hypertension, providing valuable insights for further research, personalized treatment approaches, and the advancement of precision medicine in the management of hypertension and related comorbidities.

## Data availability statement

The original contributions presented in the study are included in the article/supplementary material. Further inquiries can be directed to the corresponding authors.

## Ethics statement

The studies involving humans were approved by Ethical Review Committee (Ref. No. GCUF/ERC/39) of Government College University Faisalabad (GCUF). The studies were conducted in accordance with the local legislation and institutional requirements. The participants provided their written informed consent to participate in this study.

## Author contributions

MSHA contributed to project administration, conceptualization, study design and manuscript writing. MS and SS contributed to formal analysis, sample detection, data processing and literature search. KR contributed to conceptualization, study design, data curation, revising it critically for intellectual content. AN and TM contributed to manuscript review, data processing and interpretation of the data. All the authors agreed with the final approval of the version to be published and accountable for all aspects of the work.
